# Alcohol consumption among adults in Germany: heavy episodic drinking

**DOI:** 10.17886/RKI-GBE-2017-045

**Published:** 2017-06-14

**Authors:** Cornelia Lange, Kristin Manz, Benjamin Kuntz

**Affiliations:** Robert Koch Institute, Department for Epidemiology and Health Monitoring, Berlin, Germany

**Keywords:** ALCOHOL, HEAVY EPISODIC DRINKING, ADULTS, HEALTH MONITORING, GERMANY

## Abstract

Consuming harmful amounts of alcohol is considered a contributing factor in over 200 diseases. Heavy episodic drinking is a particularly risky drinking pattern, with possible consequences such as acute alcohol intoxication, injuries and violence. GEDA 2014/2015-EHIS defines heavy episodic drinking as the consumption of six or more alcoholic beverages on one occasion at least once per month. 24.9% of women and 42.6% of men show this drinking pattern at least once per month. Regular heavy episodic drinking is most common among 18- to 29-year-olds. The prevalence of heavy episodic drinking among highly educated women (all age groups) and men (aged over 45) is lower than it is among those with lower levels of education. The prevention of harmful levels of alcohol consumption requires measures including social and environmental interventions as well as targeting the individual behaviour which are focused on young adults as well as the diverse drinking patterns seen among groups with different levels of education.

## Introduction

Alcohol is a potentially addictive psychoactive substance. Consuming harmful levels of alcohol is considered a contributing factor to over 200 diseases; globally, it is among the five key risk factors for disease, impairment and death [1]. In addition, to the impacts harmful amounts of alcohol can potentially have on a person’s health, the World Health Organization (WHO) also highlights the socioeconomic costs for individuals who drink and the consequences for others and society in general [1]. For society, the consequences of people consuming harmful levels of alcohol include the direct costs to the health system, as well as the costs related to the loss of productivity and immaterial costs such as the loss of quality of life. In Germany, alcohol consumption is estimated to cost the economy around EUR 40 billion annually, with around one quarter of this sum being spent directly on the health care system [2, 3].

Heavy episodic drinking is a drinking pattern which poses a particularly high risk to an individual’s health and can lead to acute alcohol intoxication, injuries and violence. On the long-term, it can lead to addiction and damages to multiple organs [4]. Such damage can occur even if a person’s alcohol consumption is, on average, relatively low [1]. To reduce the population’s consumption of harmful levels of alcohol, the WHO has developed global and European strategies [5, 6]. The WHO Global Action Plan for the Prevention and Control of Non-communicable Diseases strives for a relative 10% reduction of harmful drinking patterns by 2025 (with 2010 levels as a benchmark) [7]. In part, the WHO strategy guides Germany’s national health target ‘Reduce alcohol consumption’, which was initially published in 2015 [8].

**GEDA 2014/2015-EHIS**

**Data holder:** Robert Koch Institute

**Aims:** To provide reliable information about the population’s health status, health-related behaviour and health care in Germany, with the possibility of a European comparison

**Method:** Questionnaires completed on paper or online

**Population:** People aged 18 years and above with permanent residency in Germany

**Sampling:** Registry office sample; randomly selected individuals from 301 communities in Germany were invited to participate

**Participants:** 24,016 people (13,144 women; 10,872 men)

**Response rate:** 26.9%

**Study period:** November 2014 - July 2015

**Data protection:** This study was undertaken in strict accordance with the data protection regulations set out in the German Federal Data Protection Act and was approved by the German Federal Commissioner for Data Protection and Freedom of Information. Participation in the study was voluntary. The participants were fully informed about the study’s aims and content, and about data protection. All participants provided written informed consent.

More information in German is available at www.geda-studie.de

## Indicator

Heavy episodic drinking (HED) is defined as the intake of 60 g or more of pure alcohol at a single occasion at least once per month [1]. This is the equivalent to six standard drinks containing roughly 10 g of pure alcohol per glass. To assess the frequency and amounts of alcohol people consume, the German Health Update 2014/2015-EHIS (GEDA 2014/2015-EHIS) survey used the instruments provided by the European Health Interview Survey (EHIS) [9]. To assess HED, the survey asked: ‘In the past 12 months, how often have you had 6 or more drinks containing alcohol on one occasion? For instance, during a party, a meal, an evening out with friends, alone at home, …’. To calculate the indicator, the nine possible answers were condensed into four categories: (1) at least once a week (every day or almost every day, 5-6 days a week, 3-4 days a week, 1-2 days a week); (2) every month (on 2-3 days per month, once a month); (3) less than once a month; and (4) never (not in the past 12 months, never in my whole life). Furthermore, the category of at least monthly heavy episodic drinking was established that combines the categories at least once a week and every month. The results are stratified based on gender, age and education, and for at least monthly heavy episodic drinking, according to gender and German federal state. Statistically, where confidence intervals do not overlap, the survey assumes significant differences between groups.

The analyses are based on the data received from 23,704 respondents aged 18 and above (12,953 women, 10,751 men) who gave valid answers on heavy episodic drinking. Calculations were carried out using a weighting factor that corrects for deviations within the sample from the German population (as of 31 December 2014) with regard to gender, age, district type and education. The district type accounts for the degree of urbanisation and reflects the regional distribution in Germany. The International Standard Classification of Education (ISCED) was used to ensure that the responses provided on educational levels were comparable [10]. A detailed description of the methodology applied in the GEDA 2014/2015-EHIS study can be found in the article German Health Update: New data for Germany and Europe in issue 1/2017 of the Journal of Health Monitoring.

## Results and discussion

48.8% of women and 29.7% of men responded that they had never, or at least not during the past 12 months, drunk six or more alcoholic beverages on a single occasion. 24.9% of women and 42.6% of men (Table 1 and Table 2) said they engaged in heavy episodic drinking at least once per month. Monthly heavy episodic drinking is most widespread among 18- to 29-year-olds (women 35.6%, men 54.3%). In the other age groups, no notable differences in the prevalence of heavy episodic drinking among women exist. In the older age groups (≥ 30 years), around one fifth of women drink six alcoholic beverages or more on a single occasion at least once per month. For men, the prevalence of heavy episodic drinking drops with age; yet still over one third of men (35.1%) aged 65 and over engage in heavy episodic drinking at least once per month.

Across all age groups, there are fewer highly educated women who drink six or more alcoholic beverages in a single session than women with a lower or medium level of education. For men in the 18-44 age group, no such association with education appears to exist. For men over 45, the prevalence of heavy episodic drinking among those highly educated is lower than among the group with a lower level of education. Compared to figures for the consumption of risky amounts, the figures for heavy episodic drinking reveal an inverse education gradient. These results are in line with other surveys that indicate the higher prevalence of risky drinking patterns, such as heavy episodic drinking, in socially disadvantaged groups [11]. Moreover, drinking the same amount of alcohol can cause more severe health problems in socioeconomically deprived population groups than among privileged groups, an effect known as the alcohol harm paradox [11]. As alcohol poses a greater risk to women and men with lower levels of education in the long term [12], preventive measures should be specifically focused on these groups.

**Figure 1:**
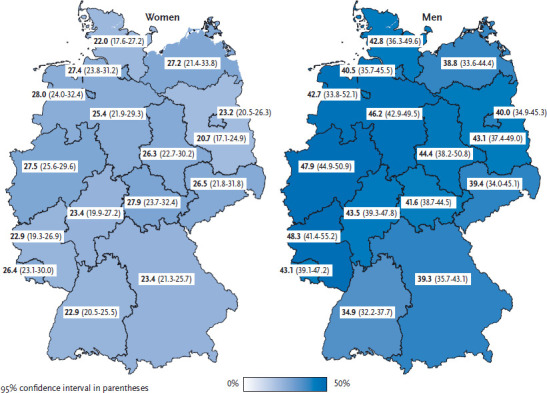
Heavy episodic drinking according to gender and federal state (n=12,953 women; n=10,751 men) Source: GEDA 2014/2015-EHIS

Compared to the German average, the prevalence of heavy episodic drinking among men is significantly higher in North Rhine-Westphalia and significantly lower in Baden-Württemberg. For women, variance between federal states and in comparison with the German average is low. The prevalence of heavy episodic drinking among women is lowest in Brandenburg (20.7%) and significantly higher in North Rhine-Westphalia (27.5%) (Figure 1).

The GEDA 2014/2015-EHIS figures are thereby notably higher than those surveyed in the German Health Interview and Examination Survey for Adults (DEGS1) [13] and GEDA 2012 [14]. As data collection methods and survey instruments have changed since DEGS1 and earlier GEDA waves, the data cannot be used to calculate trends. Nonetheless, the data consistently show that heavy episodic drinking is most common in the 18 to 29 age group. Recent data from the Epidemiological Survey of Substance Abuse also reveal a similar age pattern [15]. This highlights the need to offer measures including social and environmental interventions as well as targeting the individual behaviour of this age group. Such measures would need to consider the diverse consumption patterns according to education levels. The campaign ‘Kenn dein Limit – Eine Kampagne für Jugendliche zum Thema verantwortungsbewusster Umgang mit Alkohol’ (Respect your limits – a responsible drinking campaign for young people, http://www.kenn-dein-limit.info/) run by Germany’s Federal Centre for Health Education (BZgA) is targeted specifically at adolescents and young adults. For adults, the campaign ‘Kenn dein Limit – Bewusst genießen, im Limit bleiben’ (Respect your limits – enjoy responsibly and stay below the limit) provides information and recommendations for low-risk drinking (https://www.kenn-dein-limit.de/).

## Key statements

- 25% of women and 43% of men engage in heavy episodic drinking at least once per month.
- Among 18- to 29-year-olds, more than half of all men and over a third of all women drink six or more units on one occasion at least once per month.
- For those aged 65 or over, more than one third of men and around one fifth of women engage in heavy episodic drinking at least once per month.
- As a behavioural pattern, heavy episodic drinking is less frequent among highly educated women than among those with lower levels of education. In men, the same pattern is shown for those aged over 45.

## Figures and Tables

**Table 1:** Heavy episodic drinking among women according to age and educational status (n=12,953) Source: GEDA 2014/2015-EHIS

Women	Never (in the past 12 months)		Less than once a month		Every month		At least once week		At least monthly heavy episodic drinking	
	%	(95% CI)	%	(95% CI)	%	(95% CI)	%	(95% CI)	%	(95% CI)
**Women total**	**48.8**	**(47.5-50.0)**	**26.4**	**(25.4-27.3)**	**19.2**	**(18.3-20.1)**	**5.7**	**(5.3-6.2)**	**24.9**	**(23.9-25.8)**
**18-29 Years**	32.9	(30.3-35.7)	31.4	(28.9-34.1)	28.6	(26.0-31.4)	7.0	(5.6-8.6)	35.6	(32.9-38.4)
Low education	40.7	(34.1-47.7)	23.8	(18.2-30.6)	26.0	(20.3-32.5)	9.5	(6.2-14.2)	35.4	(29.2-42.1)
Medium education	29.5	(26.5-32.6)	32.9	(29.8-36.1)	30.9	(27.8-34.1)	6.8	(5.3-8.7)	37.7	(34.4-41.0)
High education	34.4	(29.3-39.9)	37.8	(32.7-43.2)	23.7	(19.3-28.6)	4.1	(2.6-6.4)	27.8	(23.1-33.0)
**30-44 Years**	47.2	(44.7-49.6)	30.8	(28.8-32.9)	17.6	(15.9-19.5)	4.4	(3.6-5.4)	22.0	(20.0-24.2)
Low education	46.2	(38.9-53.6)	32.2	(25.8-39.4)	17.8	(13.0-24.0)	3.8	(1.9-7.6)	21.6	(16.1-28.4)
Medium education	43.8	(40.8-46.8)	31.4	(28.9-34.0)	19.7	(17.3-22.3)	5.2	(4.0-6.7)	24.9	(22.2-27.8)
High education	55.9	(52.1-59.7)	28.8	(25.5-32.4)	12.5	(10.5-14.8)	2.8	(1.9-4.0)	15.3	(13.1-17.6)
**45-64 Years**	50.2	(48.3-52.0)	26.0	(24.5-27.5)	18.3	(16.9-19.7)	5.6	(4.9-6.4)	23.9	(22.4-25.5)
Low education	47.1	(42.8-51.4)	23.5	(20.0-27.5)	20.7	(17.6-24.3)	8.6	(6.4-11.5)	29.3	(25.5-33.5)
Medium education	50.1	(47.8-52.5)	26.3	(24.4-28.2)	18.3	(16.5-20.2)	5.3	(4.4-6.4)	23.6	(21.7-25.7)
High education	52.9	(50.2-55.7)	27.2	(24.8-29.6)	16.0	(14.0-18.3)	3.9	(2.9-5.2)	19.9	(17.8-22.3)
**≥ 65 Years**	58.0	(55.8-60.3)	20.1	(18.4-21.9)	15.8	(14.2-17.5)	6.1	(5.1-7.3)	21.9	(20.1-23.8)
Low education	60.9	(57.1-64.6)	16.0	(13.4-19.0)	17.3	(14.7-20.2)	5.8	(4.0-8.2)	23.1	(19.9-26.6)
Medium education	54.5	(51.3-57.8)	23.5	(21.0-26.2)	15.3	(13.2-17.8)	6.7	(5.2-8.4)	22.0	(19.4-24.8)
High education	64.8	(59.9-69.3)	19.6	(16.1-23.7)	10.8	(8.1-14.3)	4.8	(3.2-7.0)	15.6	(12.4-19.5)
**Total (women and men)**	**39.4**	**(38.4-40.4)**	**27.0**	**(26.4-27.7)**	**23.7**	**(22.9-24.5)**	**9.8**	**(9.4-10.3)**	**33.5**	**(32.7-34.4)**

CI=confidence interval

**Table 2:** Heavy episodic drinking among men according to age and educational status (n=10,751) Source: GEDA 2014/2015-EHIS

Men	Never (in the past 12 months)		Less than once a month		Every month		At least once week		At least monthly heavy episodic drinking	
	%	**(95% CI)**	%	(95% CI)	%	(95% CI)	%	(95% CI)	%	(95% CI)
**Men total**	**29.7**	**(28.5-31.0)**	**27.7**	**(26.6-28.8)**	**28.4**	**(27.3-29.6)**	**14.1**	**(13.3-15.0)**	**42.6**	**(41.2-43.9)**
**18-29 Years**	19.9	(17.4-22.6)	25.9	(23.4-28.5)	33.9	(31.2-36.8)	20.4	(17.8-23.1)	54.3	(51.2-57.3)
Low education	26.3	(20.4-33.1)	22.6	(17.4-28.8)	25.1	(19.4-31.9)	26.0	(20.0-33.0)	51.1	(44.2-58.0)
Medium education	18.6	(15.8-21.7)	26.6	(23.1-30.3)	36.4	(33.1-39.9)	18.4	(15.5-21.7)	54.8	(51.1-58.6)
High education	13.9	(9.8-19.2)	27.9	(22.5-34.0)	40.1	(33.9-46.6)	18.2	(14.0-23.3)	58.3	(51.4-64.8)
**30-44 Years**	26.0	(23.8-28.3)	30.9	(28.6-33.3)	31.4	(29.1-33.7)	11.8	(10.1-13.6)	43.1	(40.6-45.7)
Low education	35.7	(28.4-43.8)	20.5	(14.6-27.8)	26.0	(19.5-33.8)	17.8	(12.1-25.4)	43.8	(35.8-52.2)
Medium education	24.5	(21.6-27.6)	31.9	(28.7-35.3)	32.1	(29.1-35.2)	11.5	(9.4-14.0)	43.6	(40.2-47.1)
High education	24.9	(21.9-28.0)	32.9	(29.6-36.3)	32.5	(29.1-36.1)	9.8	(7.7-12.3)	42.3	(38.4-46.3)
**45-64 Years**	30.2	(28.4-32.0)	28.8	(27.1-30.6)	27.5	(25.7-29.3)	13.5	(12.3-14.9)	41.0	(39.0-43.0)
Low education	32.2	(27.8-37.0)	19.9	(16.5-23.8)	26.3	(22.2-30.7)	21.7	(17.8-26.1)	47.9	(43.0-52.9)
Medium education	29.8	(27.5-32.3)	28.6	(26.2-31.1)	27.7	(25.3-30.2)	13.9	(12.2-15.8)	41.6	(38.9-44.3)
High education	30.2	(27.8-32.7)	32.3	(30.1-34.7)	27.4	(25.0-29.9)	10.1	(8.6-11.8)	37.5	(34.9-40.0)
**≥ 65 Years**	40.8	(38.4-43.3)	24.1	(22.2-26.1)	22.5	(20.8-24.2)	12.6	(11.2-14.1)	35.1	(33.0-37.2)
Low education	37.3	(32.2-42.8)	21.9	(18.0-26.5)	24.6	(20.0-29.9)	16.2	(12.4-20.7)	40.7	(35.5-46.2)
Medium education	40.7	(37.0-44.5)	23.5	(20.7-26.6)	22.8	(20.1-25.6)	13.0	(11.2-15.2)	35.8	(32.8-38.9)
High education	43.0	(39.8-46.3)	26.2	(23.2-29.5)	20.7	(18.3-23.3)	10.1	(8.4-12.1)	30.8	(28.0-33.7)
**Total (women and men)**	**39.4**	**(38.4-40.4)**	**27.0**	**(26.4-27.7)**	**23.7**	**(22.9-24.5)**	**9.8**	**(9.4-10.3)**	**33.5**	**(32.7-34.4)**

CI=confidence interval
